# Particle embolization to control life-threatening hemorrhage from a fungating locally advanced breast carcinoma: a case report

**DOI:** 10.1186/1752-1947-6-186

**Published:** 2012-07-04

**Authors:** John M Moriarty, Minzhi Xing, Christopher T Loh

**Affiliations:** 1 Department of Radiological Sciences, David Geffen School of Medicine at UCLA, Los Angeles, CA, USA; 2 Sydney Medical School, University of Sydney, Sydney, NSW, Australia

## Abstract

**Introduction:**

Sudden severe hemorrhage from locally advanced fungating breast carcinoma and its associated cutaneous lesions is rarely reported. Transcatheter arterial embolization has been used widely in the setting of intractable neoplastic hemorrhage arising from primary and metastatic tumors of the lung, liver, kidney, and gastrointestinal tract. Here, we detail the use of transcatheter arterial embolization in controlling torrential hemorrhage in a patient with advanced invasive breast cancer and multiple comorbidities.

**Case presentation:**

We report the case of a 28-year-old African-American woman who presented with acute torrential hemorrhage from a high-grade invasive ductal breast carcinoma. A computed tomography scan demonstrated a 14cm mass with extensive muscle, fascial, and cutaneous invasion. Owing to the extent of invasion and multiple comorbidities, she was deemed to be unsuitable for surgical management. Selective angiography of the left internal mammary artery revealed no tumoral blush, extravasation, or pseudoaneurysm. Transcatheter arterial embolization was undertaken, and complete occlusion of the vessel was demonstrated. No further episodes of hemorrhage occurred.

**Conclusions:**

Though rare, sudden severe hemorrhage from advanced breast cancer may be definitively managed by embolization alone and thus surgery may be avoided.

## Introduction

Sudden severe hemorrhage from locally advanced fungating breast carcinoma and its associated cutaneous lesions is rarely reported. Transcatheter arterial embolization (TAE) has been used widely in the setting of intractable neoplastic hemorrhage arising from primary and metastatic tumors of the lung, liver, kidney, and gastrointestinal tract [1]. Embolization of the internal mammary artery (IMA) via a similar approach has been described for blunt chest trauma [2]. Here, we detail the use of TAE in controlling torrential hemorrhage in a patient with advanced invasive breast cancer and multiple comorbidities.

## Case presentation

A 28-year-old African-American woman was admitted for emergent management of acute hemorrhage from a left breast mass. One year earlier, she had sought medical attention for a palpable breast lump, which was subsequently diagnosed as a high-grade invasive ductal breast carcinoma. Her medical care had been complicated by an initial delay in diagnosis of two months, followed by a decision by the patient to pursue alternative and herbal medical therapies rather than the mastectomy and adjuvant chemotherapy originally suggested by her surgical oncologist.

**Figure 1:**
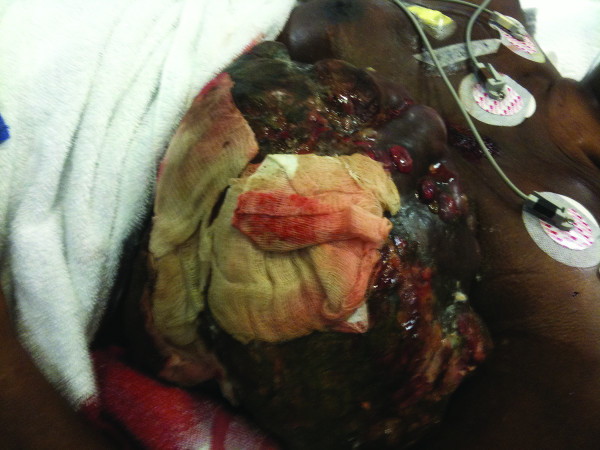
Photograph of the left side of the thorax demonstrates an extremely large, fungating breast carcinoma with extensive cutaneous ulceration.

She re-presented several months after the original diagnosis with a very large (14cm), locally aggressive left breast mass (Figure 1) that had invaded the overlying skin and underlying musculature, and multidrug-resistant polymicrobial cellulitis was concurrently diagnosed. By this time, she had failed initial chemotherapy and was being managed on ixabepilone alone. Furthermore, she had developed *Clostridium difficile* pseudomembranous colitis and was on low-molecular-weight heparin therapy for a right upper limb deep vein thrombosis. She had no previous episodes of hemorrhage. After her presentation, the breast mass began to weep acutely from multiple cutaneous sites. Our patient became rapidly unstable with hypotension, tachycardia, alteration in level of consciousness, and a decrease in hemoglobin levels to 6.1mg/dL. Fluid and blood product resuscitations were immediately started, and our patient was transferred to the intensive care unit (ICU). A surgical review found no suitable surgical option, and she was transferred emergently to the angiography suite for management by interventional radiology (IR).

**Figure 2:**
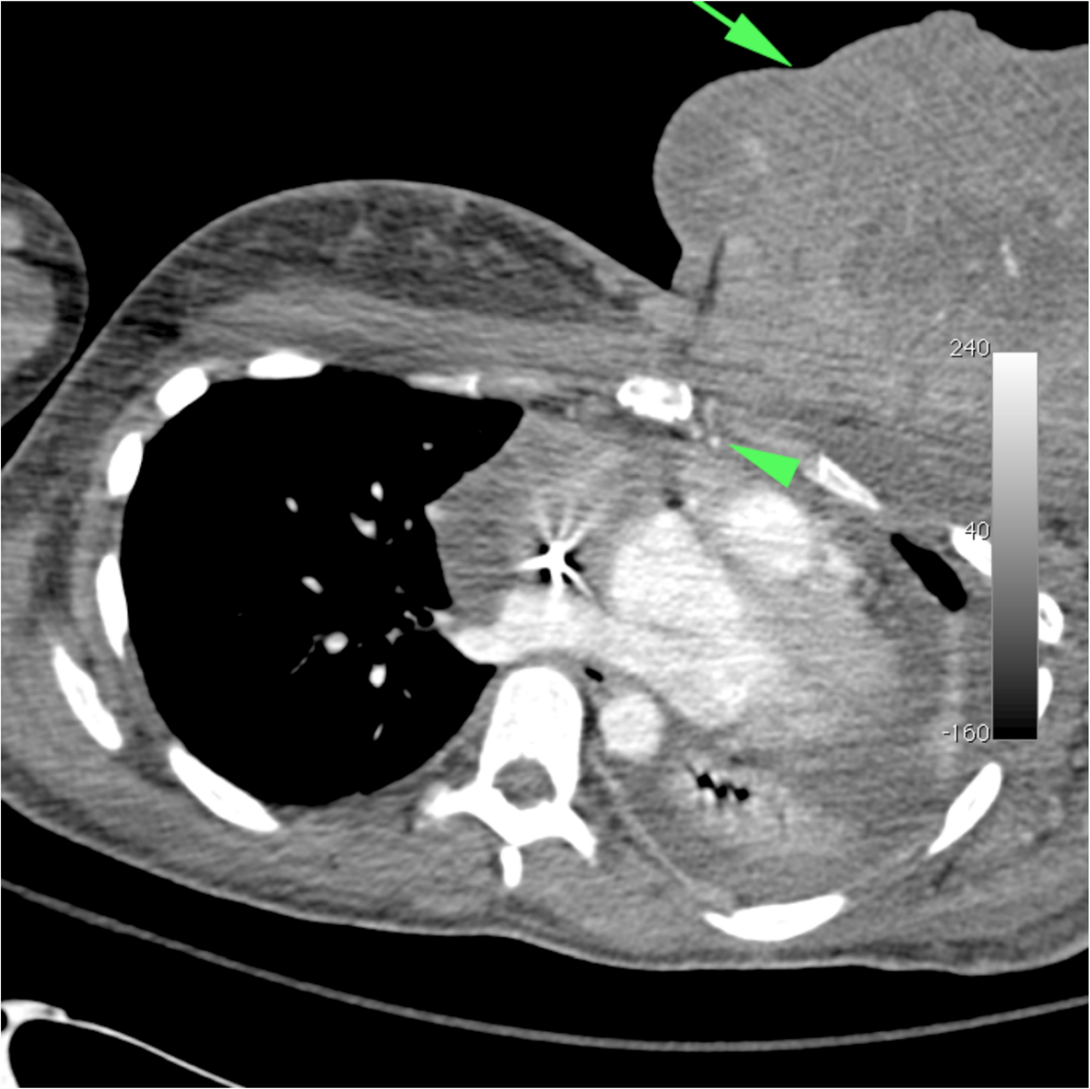
**Axial contrast-enhanced computed tomography image of the thorax demonstrates a 14cm enhancing breast mass (arrow).** Branch vessels of the left internal mammary artery (arrowhead) passing anteriorly into the mass were noted.

**Figure 3:**
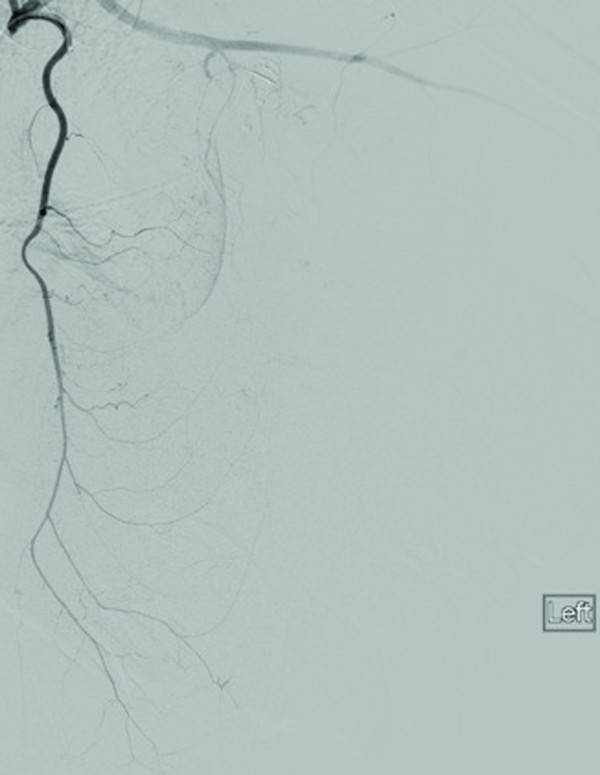
Selective digital subtraction angiogram of the left internal mammary artery shows multiple feeding branches to the anterior chest wall and left breast.

**Figure 4:**
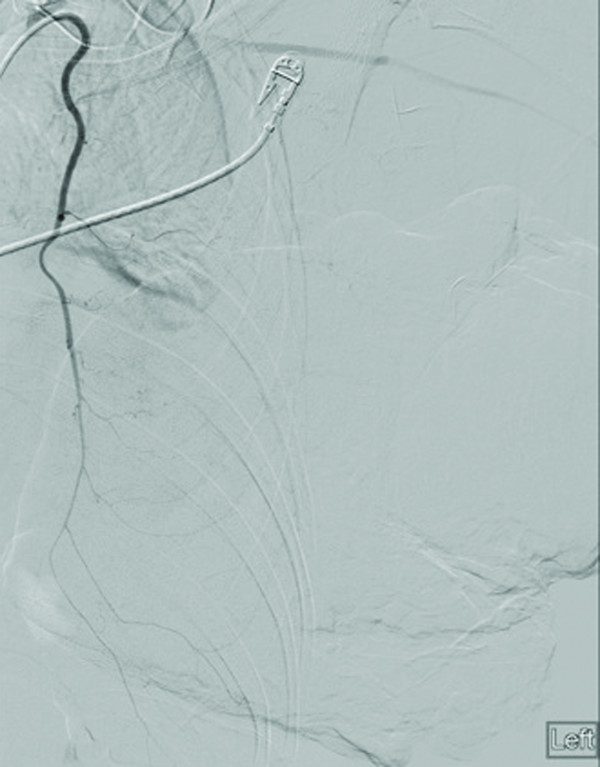
Pre-particle embolization images of the left internal mammary artery demonstrate proximal occlusion of the vessel and its mammary branches.

**Figure 5:**
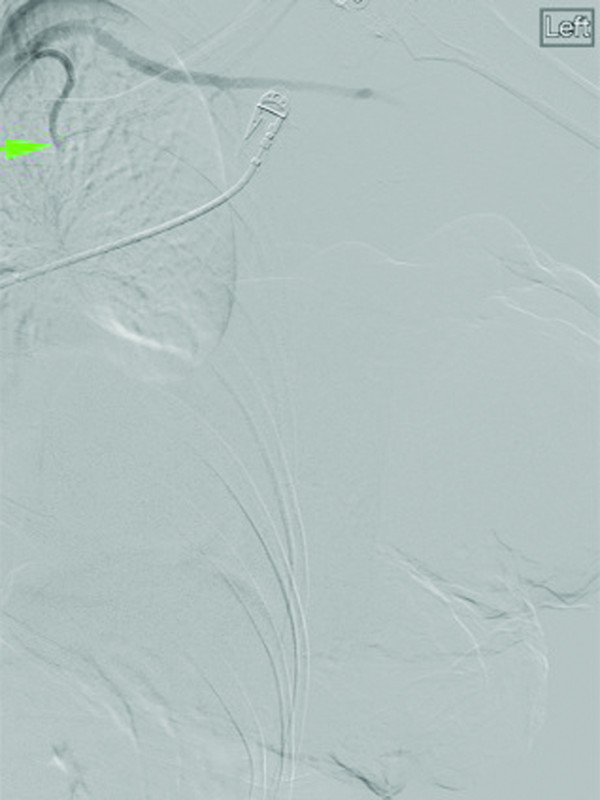
Post-particle embolization images of the left internal mammary artery demonstrate proximal occlusion of the vessel and its mammary branches.

**Figure 6:**
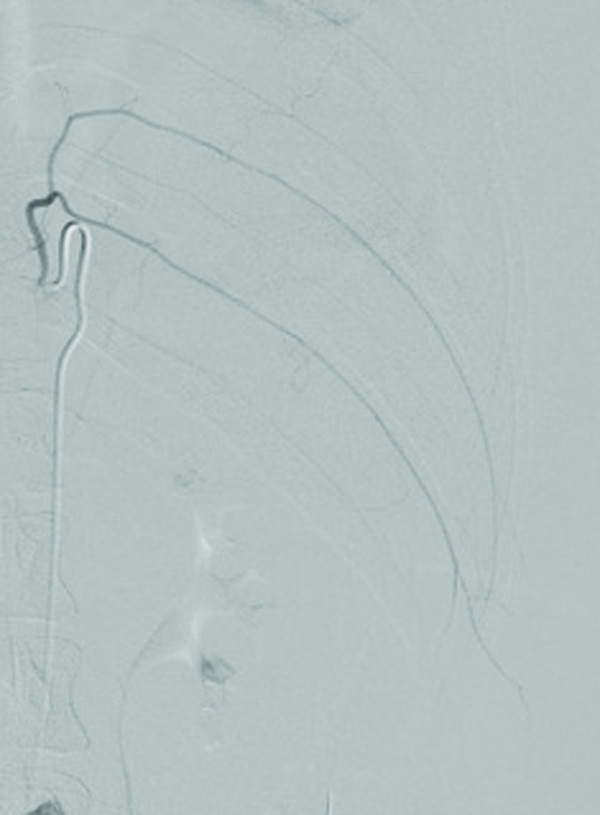
**Selective right intercostal artery angiography was performed through a 5-French Michelson catheter from the supreme right intercostal artery to the right 12th intercostal artery.** A common trunk of the right ninth and 10th intercostal arteries is pictured. No discrete bleeding source or arterial irregularity was identified.

**Figure 7:**
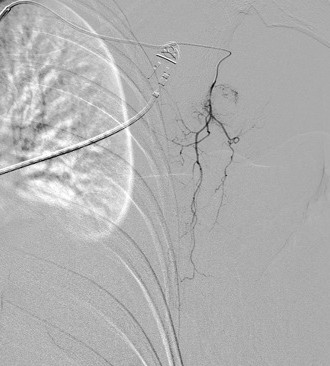
Selective injection of the left lateral thoracic artery failed to demonstrate any discrete communication with the breast mass or bleeding site.

A review of a pre-existing contrast-enhanced computed tomography (CT) scan demonstrated a large (14cm) left breast mass with extensive local muscle, fascial, and cutaneous invasion (Figure 2). Prominent branches of the left IMA passing anteriorly to the mass were also noted. Selective angiography of the left IMA by means of a 6-French internal mammary guiding catheter revealed no discrete tumoral blush, extravasation, or pseudoaneurysm (Figure 3). However, given the CT findings, embolization using 300 to 500μm and 500 to 700μm microspheres (Embosphere®; BioSphere Medical, South Jordan, UT, USA) through a 4-French angled hydrophilic catheter was undertaken, and complete occlusion of the vessel was demonstrated (Figures 4 and 5). The results of subsequent selective angiography of the left intercostal (Figure 6), lateral thoracic (Figure 7), and dorsal scapular arteries were unremarkable. An evaluation of the left inferior epigastric artery revealed no communication with the breast mass or ulceration.

By this time, while the breast mass continued to ooze blood, the frank hemorrhage from the skin site had slowed, and a decision to halt investigation and return our patient to the ICU was reached. Over the next 12 hours, the bleeding ceased and our patient returned to baseline. At two-month follow up, she had no further episodes of hemorrhage.

## Discussion

Locally advanced breast cancers (LABCs) currently account for 10% of breast cancer in women [3], and the subset of synchronous stage IV metastatic disease occurs in 3% to 4% of all diagnoses. Prognosis in patients with LABC has historically been very poor, and there is an extremely high risk of local recurrence and distant metastases despite aggressive surgical intervention and primary radiation therapy [4]. The additions of post-operative radiotherapy to surgery and of neoadjuvant systemic therapies to local therapies have further improved local control and disease-free survival. The occurrence of severe spontaneous bleeding with LABC is rare and has been described in several case reports, in which it was reported to result from a rupture of tumor vessels adjacent to concomitant cysts [5], cartilaginous metaplasia and intra-tumor bleeding [6], and intra-ductal hematoma formation with diffuse dissecting hemorrhage throughout the surrounding breast tissue [7]. In addition, reports by Tokizawa and colleagues [8] and Nagasawa and colleagues [9] describe sudden hemorrhage due to breast cancer localized to the mammary glands in the absence of skin invasion.

The main vasculature to the breast consists of the second to fifth internal mammary superomedial perforators that arise from the IMA and that provide approximately 60% of its total blood supply. The thoracoacromial artery, vessels to the serratus anterior, lateral thoracic artery, and the terminal branches of the third to eighth intercostal perforators serve as the remainder of the vascular supply to the region. The IMA itself arises directly from the subclavian artery and courses down the chest wall anterior to the pleura and endothoracic fascia, terminating at the sixth intercostal space as the musculophrenic and superior epigastric arteries. Flow rates of up to 240mL per minute through the IMA potentially allow 1litre of blood loss within several minutes, and massive hemothorax, hemomediastinum, or pericardial tamponade may result. The mechanism of sudden-onset hemorrhage from a completely transected IMA has been discussed in the trauma setting, where vessel retraction into the pectoralis muscle and hemostasis may occur as a result of arterial spasm and hypotension [10]. Resuscitation and resolution of arterial spasm may then lead to severe delayed-onset bleeding.

Surgical ligation of the IMA via median sternotomy or lateral thoracotomy is an option for stemming massive hemorrhage originating from the IMA perforators [2]. Operative outcomes of IMA ligation in breast cancer have not been characterized. However, intra-operative difficulty in locating and controlling bleeding vessels may occur, especially in the presence of large, friable, and highly vascularized tumor masses. Owing to the extensive mass and degree of local invasion in the present case, surgical intervention was deemed not appropriate.

Catheter-directed minimally invasive therapies for management of hemorrhage have evolved from their earliest form with the use of autologous clot to include many different forms of embolic agents, such as particles, liquid embolic agents such as glue, and coils. Furthermore, embolization, typically performed by IR, has evolved to include management of hemorrhage in almost every vascular territory, including the brain, lungs, liver, gastrointestinal tract, uterus, and peripheries.

The role of IR in cancer has progressed to include definitive therapy for control of bleeding. In the setting of severe and recurrent hemorrhage from metastatic breast carcinoma, Harrington and colleagues [4] and Rankin and colleagues [2] described TAE for successful hemostasis in two out of three and seven out of nine patients, respectively, by using mixtures of autologous blood clot, Gelfoam® (Pfizer Inc, New York, NY, USA), Ivalon® (Fabco, New London, CT, USA), steel coils, and dextrose 50%. In one patient, partial recanalization of the artery occurred with simultaneous development of transient tunnel vision, thought to be due to backflow of autologous clot from the IMA to the adjacent vertebral artery [4]. Although secure placement of the catheter in the vessel helps to reduce this complication, it is noted that, owing to pulsatile flow in the artery, the risk cannot be completely eliminated. Hence, the use of permanent agents such as particles, coils, and liquid embolics is more desirable in the setting of acute hemorrhage. In addition, although Gelfoam® is known to be excellent in controlling acute hemorrhage with minimal tissue ischemia, a previous report has noted that catheterization of the IMA may require the use of a catheter too small in diameter to permit injection of Gelfoam® pledgets [10]. In our case, the use of microspheres was noted to be sufficient to achieve complete occlusion of the IMA and to prevent recurrent episodes of hemorrhage.

Besides embolization of feeding arteries, IR techniques for LABC include the use of transcatheter arterial chemoembolization to achieve both embolization of the tumor and delivery of chemotherapy drug-eluting microspheres. Morimoto and colleagues [11] first described the technique of mixing anticancer agents and clotting factors for selective injection into the IMA and lateral thoracic and thoracodorsal arteries as a pre-surgical treatment. Macroscopic regression of primary tumors in all patients was observed, and there were no systemic toxic effects of chemotherapy. Takaziwa and colleagues [12] described the use of redistributed subclavian arterial infusion chemotherapy, in which an implanted catheter-port system was used after redistribution of arterial blood supply to the tumor site to overcome problems related to the formation of collateral arterial blood supplies in arterial infusion chemotherapy. This was noted to be especially useful for patients who had multiple comorbidities and who were otherwise unable to physically tolerate chemotherapy. Other minimally invasive options for LABC include thermal breast ablation techniques such as radiofrequency, high-intensity focused ultrasonography and cryoablation. In particular, radiofrequency ablation has been shown to be effective and feasible for minimally invasive treatment of non-operable older patients with early-stage, primary breast carcinoma [13]. Cryotherapy has also been found to be useful in reducing tumor margins and to have good cosmesis and no short-term local recurrences [14].

## Conclusions

Sudden severe hemorrhage from advanced breast carcinoma is a rare phenomenon that presents a management dilemma in the presence of multiple comorbidities. Angiography and transcatheter embolization allow rapid and durable cessation of hemorrhage and provide definitive treatment with minimal risk where conventional treatment modalities fail or are too morbid to perform.

## Consent

Written informed consent was obtained from the patient for publication of this case report and any accompanying images. A copy of the written consent is available for review by the Editor-in-Chief of this journal.

## Abbreviations

CT, Computed tomography; ICU, Intensive care unit; IMA, Internal mammary artery; IR, Interventional radiology; LABC, Locally advanced breast cancer; TAE, Transcatheter arterial embolization.

## Competing interests

The authors declare that they have no competing interests.

## Authors’ contributions

JMM helped to perform the angiographic evaluation and interventional procedures on the patient and was a major contributor in writing and editing the case presentation and literature review. CTL helped to perform the angiographic evaluation and interventional procedures on the patient. MX was a major contributor in writing and editing the case presentation and literature review*.* All authors read and approved the final manuscript.

## References

[B1] Lin SC Shih SC Kao CR Chou SY Transcatheter arterial embolization treatment in patients with hepatocellular carcinoma and risk of pulmonary metastasis World J Gastroenterol 2003 9 1208 1211 12800225 10.3748/wjg.v9.i6.1208 PMC4611785

[B2] Rankin EM Rubens RD Reidy JF Transcatheter embolisation to control severe bleeding in fungating breast cancer Eur J Surg Oncol 1988 14 27 32 3345852

[B3] Newman LA Epidemiology of locally advanced breast cancer Semin Radiat Oncol 2009 19 195 203 10.1016/j.semradonc.2009.05.003 19732683

[B4] Harrington DP Barth KH Baker RR Truax BT Abeloff MD White RI Therapeutic embolization for hemorrhage from locally recurrent cancer of the breast Radiology 1978 129 307 310 704842 10.1148/129.2.307

[B5] Maekawa S Shimoda K Furuyama M Ikejiri K A case report of breast cancer concomitant with huge cyst due to rapture of the tumor vessels J Jpn Surg Assoc 1992 53 2091 2094 In Japanese with English Summary

[B6] Wada N Ikeda T Hiramatus H A case of breast cancer which rapidly grew up to a huge mass because of intratumor bleeding J Jpn Soc Clin Surg 1996 57 2951 2954 In Japanese with English summary

[B7] Miller WL Armstrong AL Diffuse hemorrhage of the breast caused by underlying carcinoma of the breast: a case report J Ky Med Assoc 1987 85 600 602 3681121

[B8] Tokizawa N Iino Y Yokoe T Izumi M Kawate S Anzai T Morishita Y Honma M Sudden hemorrhage of the breast caused by breast cancer without skin invasion: report of a case Surg Today 1995 25 920 922 10.1007/BF00311760 8574061

[B9] Nagasawa M Iino Y Horiguchi J Takei H Maemura M Horii Y Matsumoto H Nagaoka H Oyama T Nakajima T Morishita Y Sudden hemorrhage of the breast caused by breast cancer: a case report and review of the literature Breast Cancer 2000 7 176 178 10.1007/BF02967454 11029794

[B10] Patel K Kimbrell BJ Marx MV Petrone P Asensio JA Angiographic embolization of an expanding breast hematoma after blunt trauma: a novel approach to a rare injury J Trauma 2009 67 E14 E16 10.1097/TA.0b013e3180485cc4 19590299

[B11] Morimoto K Takatsuka Y Sugitachi A Miyata Y Choi S Hashimoto T Hara K Combined transcatheter arterial embolization and regional chemotherapy for locally advanced carcinoma of the breast A preliminary invest Acta Radiol Oncol 1985 24 241 245 10.3109/02841868509134394 2994374

[B12] Takizawa K Shimamoto H Ogawa Y Yoshimatsu M Yagihashi K Nakajima Y Kitanosono T Development of a new subclavian arterial infusion chemotherapy method for locally or recurrent advanced breast cancer using an implanted catheter-port system after redistribution of arterial tumor supply Cardiovasc Intervent Radiol 2009 32 1059 1066 10.1007/s00270-009-9510-1 19238484

[B13] Susini T Nori J Olivieri S Livi L Bianchi S Mangialavori G Branconi F Scarselli G Radiofrequency ablation for minimally invasive treatment of breast carcinoma. A pilot study in elderly inoperable patients Gynecol Oncol 2007 104 304 310 10.1016/j.ygyno.2006.08.049 17070572

[B14] Littrup PJ Jallad B Chandiwala-Mody P D’Agostini M Adam BA Bouwman D Cryotherapy for breast cancer: a feasibility study without excision J Vasc Interv Radiol 2009 20 1329 1341 10.1016/j.jvir.2009.06.029 19800542 PMC3865783

